# Correlates of Protection, Thresholds of Protection, and Immunobridging among Persons with SARS-CoV-2 Infection 

**DOI:** 10.3201/eid2902.221422

**Published:** 2023-02

**Authors:** David S. Khoury, Timothy E. Schlub, Deborah Cromer, Megan Steain, Youyi Fong, Peter B. Gilbert, Kanta Subbarao, James A. Triccas, Stephen J. Kent, Miles P. Davenport

**Affiliations:** The University of New South Wales, Sydney, New South Wales, Australia (D.S. Khoury, D. Cromer, M.P. Davenport);; University of Sydney, Sydney (T.E. Schlub, M. Steain, J.A. Triccas);; Fred Hutchinson Cancer Research Center, Seattle, Washington, USA (Y. Fong, P.B. Gilbert);; The University of Melbourne at the Peter Doherty Institute for Infection and Immunity, Melbourne, Victoria, Australia (K. Subbarao, S.J. Kent);; Monash University, Melbourne (S.J. Kent)

**Keywords:** COVID-19, 2019 novel coronavirus disease, coronavirus disease, severe acute respiratory syndrome coronavirus 2, SARS-CoV-2, viruses, respiratory infections, zoonoses, neutralizing antibody, vaccine efficacy, standardization, correlates of protection

## Abstract

Several studies have shown that neutralizing antibody levels correlate with immune protection from COVID-19 and have estimated the relationship between neutralizing antibodies and protection. However, results of these studies vary in terms of estimates of the level of neutralizing antibodies required for protection. By normalizing antibody titers, we found that study results converge on a consistent relationship between antibody levels and protection from COVID-19. This finding can be useful for planning future vaccine use, determining population immunity, and reducing the global effects of the COVID-19 pandemic

Determining the relationship between immune response and protection from symptomatic SARS-CoV-2 infection (i.e., COVID-19) is useful for predicting the future effectiveness of vaccines. That relationship should enable immunobridging (i.e., predicting the efficacy of candidate vaccines) that can help with approval of new or updated vaccines based on immunogenicity data, without the need for large phase 3 trials (*1*). Immunobridging is used for approval of seasonal influenza vaccines in the European Union and the United States and reduces the costs and time required to develop vaccines. In addition, defining levels of immunity required for protection from novel SARS-CoV-2 variants will be useful for predicting population-level immunity to infection and guiding public health policy on vaccination and boosting.

Several studies have shown that higher levels of neutralizing antibody are associated with immune protection from symptomatic SARS-CoV-2 infection during short-term follow-up after vaccination (*2*–*6*). Three of those studies also tried to estimate the level of protection associated with particular antibody levels by using 2 approaches to estimate the relationship between neutralizing antibody levels and vaccine efficacy (*2*–*4*) (protection curve; Table; Figure 1). Although those studies reported threshold antibody levels required for 50% or 70% protection, all found that protection changes gradually with neutralization titer and, thus, there is not a strict threshold below which persons are not protected or above which protection is achieved.

**Table:** Glossary of terms used in study of correlates of protection for SARS-CoV-2 infection

Term	Definition
Protection curve	The relationship between the measured immune response of a vaccine in a subgroup of persons and the level of protection from symptomatic infection provided by the vaccine in that subgroup compared with placebo group (protection = vaccine efficacy).
Threshold of protection	The level of immune response required to provide a specified level of protection (vaccine efficacy) from COVID-19. The 50% protective threshold is commonly reported.
Fold-of-convalescent scale	An attempt to compare different assays by normalizing titers to that of convalescing persons in the same assay. Accurate comparison requires convalescing persons to have similar infection histories.
IU/mL	A neutralization titer (or mean neutralization titer) calibrated to a World Health Organization international standard and reported in IU/mL.

**Figure 1:**
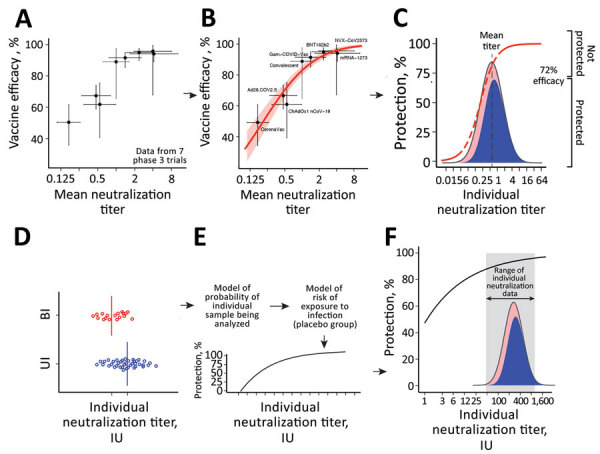
Predicting protection from symptomatic SARS-CoV-2 infection by using approaches to elucidate the relationship between neutralizing antibody titers and protection from COVID-19 (the protection curve): the vaccine-comparison (A–C) and breakthrough-infection (D–F) approaches. The 2 approaches are illustrated schematically: data used (A, D); model fit to data (B, E); and estimated protection (C, F) The vaccine-comparison approach used data on mean neutralization titer from phase 1/2 vaccine trials (normalized to convalescing persons in the same study; x-axis) and observed vaccine efficacy against symptomatic SARS-CoV-2 infection in phase 3 trials (y-axis; n = 7 vaccine trials plus 1 study of infection risk in convalescing persons) (A, B). Using the observed distribution in neutralization titers for a given vaccine and the protection curve, we sum over the whole population to predict the proportion of susceptible (red) or protected (blue) persons for a given vaccine and to estimate protective efficacy for different neutralizing antibody levels (C). Fitting across all vaccines and convalescent persons simultaneously derives the protection curve that best fits the neutralization and protection data (B). The breakthrough-infection model uses neutralization titers of persons with symptomatic breakthrough infections (n = 36 for mRNA-1273 [Moderna, https://www.modernatx.com] and n = 47 for ChAdOx1 [AstraZeneca, https://www.astrazeneca.com]) and uninfected persons (n = 1,005 for mRNA-1273 and n = 828 for ChAdOx1) (*3*,*4*). This method’s underlying risk model adjusts for demographic risk factors and for the probability of being sampled in the study to remove these potential sources of bias (E). The protection curve reflects an estimate of the vaccine efficacy in subgroups of persons with specific neutralization titers after the 2-phase sampling design was adjusted for (F). Data and model relationship in panels A and B are from (*2*).

The study of immune correlates by Khoury et al. used a vaccine-comparison approach, which estimated the relationship between mean neutralizing antibody levels (in phase 1/2 trials) and vaccine efficacy (in phase 3 trials) across 7 vaccines and convalescing persons (after first normalizing neutralization titers to convalescing persons in each study) (*2*) (Table; Figure 1, panels A–C). That study estimated that the neutralizing antibody level associated with 50% protection from COVID-19 was ≈20% of the mean titer for persons in the convalescent phase (or 54 IU/mL) (*2*). More recently, 2 studies compared neutralizing antibody titers from persons vaccinated with mRNA 1273 (Moderna, https://www.modernatx.com) or ChAdOx1 (AstraZeneca, https://www.astrazeneca.com) with or without symptomatic breakthrough infection (Figure 1, panels D–F). Those studies reported 70% protective thresholds ranging from 4 to 33 IU/mL (Table), depending on the assay used, suggesting a potential role of assay differences in the discrepancies (Appendix) (*3*,*4*). The apparent discrepancies between studies pose a challenge to the use of protection curves in guiding public health decisions. Therefore, we studied whether those results can be reconciled by accounting for the different methods used. This work was approved under the University of New South Wales Sydney Human Research Ethics Committee (approval HC200242). All data and codes are available from GitHub (https://github.com/InfectionAnalytics/ReconcilingCorrelatesOfProtection).

## Reconciling the Studies on Thresholds of Protection

A major limitation for reconciling thresholds of protection (Table) is lack of a standardized assay for measuring in vitro neutralization titers. Although an international standard has been established (*7*), reported titers seem affected by the assay used, as would be expected from differences in cells, virus, and outcomes measured (*8*). For example, even when neutralization titers are measure against the same stocks of pooled convalescent-phase plasma (e.g., the World Health Organization [WHO] 20/130 standard), different assays produced geometric mean neutralization titers (GMT) that varied from 120 to >12,000 (*7*). Even after standardizing measurements from different assays into international units (Table), standardized neutralization titers across the assays still differed by up to 50-fold (*7*). This difference in neutralization titers across different assays is also evident when comparing the 3 studies quantifying the threshold of protection (Table) (*2*–*4*). For example, Gilbert et al. reported the GMT for mRNA-1273 as ≈247 IU/mL (*4*), compared with 1,057 IU/mL reported by Khoury et al. (*2*) (Appendix). A quick survey of the literature reveals 6 reported estimates of the GMT for mRNA-1273 vaccinees, ranging from 247 IU/mL (95% CI 231–264) to 1,404 (95% CI 795–2,484) IU/mL, depending on the study (Appendix Table 1). Similarly, estimates of the GMT for ChAdOx1 vaccinees ranged from 23 IU/mL (*3*) to 144 IU/mL (*2*). When the same neutralization assay is run across different laboratories, then international standards are probably a very effective mechanism for adjusting for interlaboratory variability. However, it is clear from those discrepancies that expression of titers in international units is insufficient for normalizing between different assays and comparing the thresholds of protection reported in these studies (Appendix), which most likely results from differences in the assays themselves (*8*).

An alternative approach for normalizing neutralization titers between studies is to assume that similar groups of vaccinees should have similar titers. For example, rather than relying on conversion to the WHO international units, we can assume that the mean neutralization for the mRNA-1273 vaccinees is similar in the phase 1/2 trials (as analyzed by Khoury et al. [*2*,*10*]) and in the phase 3 trial (as analyzed by Gilbert et al. [*4*,*11*]) (Appendix). Normalization is limited because it does not account for differences in baseline characteristics of the cohort vaccinated in each study (e.g., age), which may influence neutralization titers. However, because immunobridging studies also rely on comparing vaccine titers among different groups, this approach is pragmatic for overcoming the limitations of comparing different assays.

**Figure 2:**
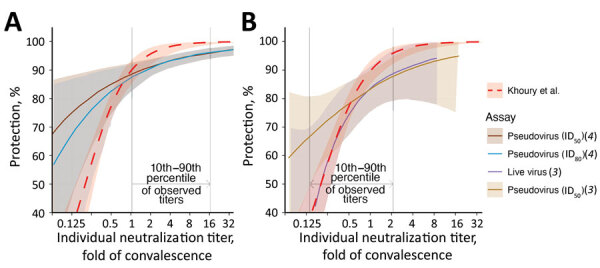
Comparisons of the estimated curves for protection from SARS-CoV-2 infection from 2 vaccines: A) mRNA-1273 (Moderna, https://www.modernatx.com) (*4*); B) ChAdOx1 (AstraZeneca, https://www.astrazeneca.com) (*3*). The relationships between vaccine efficacy against COVID-19 infection (y-axis) and neutralization titers (protection curve) that were estimated in each study (*2**–**4*) are shown. The protection curve derived from the vaccine-comparison model (red dashed line) is compared with the modeled protection curves estimated from breakthrough-infection studies by Gilbert et al. (*4**)* (dark brown for the results from the ID_50_ and teal lines for the results from the ID_80_ neutralization titer in in vitro pseudovirus neutralization assays) (A) and Feng et al. (*3*) (purple for the results from in vitro native (live) SARS-CoV-2 virus and light brown for the pseudovirus neutralization assays) (B). Shaded areas indicate 95% CIs from each model. These curves were extracted from the cited studies (Appendix, https://wwwnc.cdc.gov/EID/article/29/2/22-1422-App1.pdf), and differences between assays were controlled for by normalizing the curve from each study by the mean neutralization titer of the uninfected vaccinees in each study. The normalized curves were then represented on a fold-of-convalescent scale by multiplying by the mean neutralization titer of vaccinees compared with convalescing persons as reported in the phase 1/2 trials (*9*,*10*). The vaccine-comparison model agrees closely with the breakthrough-infection models in the neutralization titer ranges where data were most abundant (vertical gray lines indicate 10th to 90th percentiles of the data available in each study). ID_50_, 50% infectious dose; ID_80_, 80% infectious dose.

Applying this normalization approach enabled us to compare the protection curves across different immune correlate studies (Appendix). We aligned the data by assuming that the mean titer for mRNA-1273- or ChAdOx1-vaccinated persons is the same between the phase 1/2 trials and the phase 3 trials for each vaccine (Figure 2; Appendix). Although this normalization is independent of the x-axis scale used, we plotted both curves onto a fold-of-convalescent level scale (Table) developed by Khoury et al. (*2*) for illustration. This transformation enabled a more direct comparison of the protection curve across the 3 studies. Considering the mRNA-1273 breakthrough-infection model (*4*) (Figure 2, panel A), for example, we saw good agreement with the Khoury et al. model (*2*) at the higher neutralization levels achieved with mRNA-1273 vaccination (albeit a seemingly slightly lower maximum protection level predicted in the breakthrough-infection model) but very poor agreement at low neutralization levels. This finding is easily understandable considering the distribution of individual neutralization titers in the mRNA-1273 breakthrough-infection study, in which only ≈10% of participants had a neutralization titer less than the mean titer of early convalescent-phase participants (Figure 2, panel A). Thus, neutralization data with which to estimate protection at lower neutralization levels are sparse (hence, the wide confidence intervals in this region of the curve). Similarly, the ChAdOx1 protection curve (Figure 2, panel B) shows good agreement with the Khoury et al. analysis (*2*) in the region in which neutralization data are available in the breakthrough-infection study (Figure 2, panel B).

The broad CIs and divergence of the models for which neutralization data are sparse suggests the need for caution when extrapolating the relationship between neutralization and protection beyond the ranges of data available in each study. The vaccine comparison approach has the advantage of fitting to a large span of neutralization titers (a 20-fold range in GMT between the 7 vaccines) (*2*), enabling prediction of the vaccine efficacy over a wide range of neutralization titers. Because none of the reported phase 3 studies of ancestral SARS-CoV-2 infection reported efficacy <50% or >95%, the vaccine-comparison analysis also extrapolates efficacy above and below these levels. However, studies of vaccine efficacy and effectiveness against SARS-CoV-2 variants suggests that the curve remains predictive against the Alpha, Beta, Delta, and Omicron variants, for which lower neutralization titers are observed (*12*; D. Khoury et al., unpub. data, https://www.medrxiv.org/content/10.1101/2021.12.13.21267748v2).

**Figure 3:**
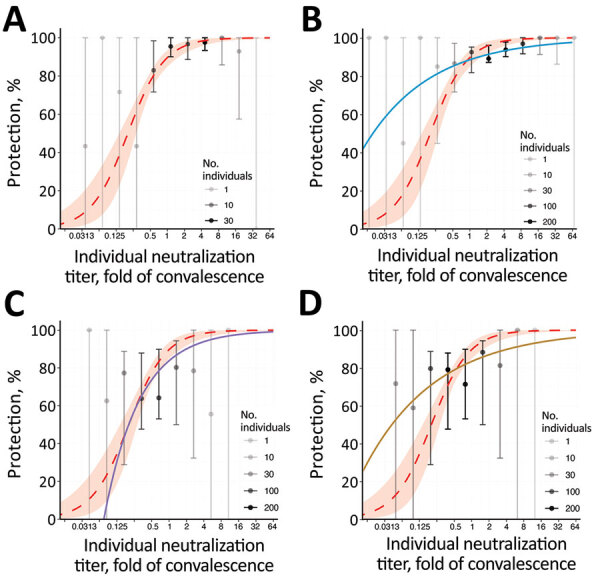
Breakthrough-infection data and protection from SARS-CoV-2 infection showing the association between neutralizing antibody titer and protection from symptomatic SARS-CoV-2 infection for an individual person. A) BNT162b2 (Pfizer-BioNTech, https://www.pfizer.com) (*5*); B) mRNA-1273 (Moderna, https://www.modernatx.com), pseudovirus ID_50_ (*4*); C) ChAdOx1 (AstraZeneca, https://www.astrazeneca.com), live virus (*3*); D) ChAdOx 1, pseudovirus ID_50_ (*3*). The protection curve derived from the vaccine-comparison model (red dashed line and shading 95% CIs) is compared with the observed normalized frequencies of neutralization level (calculations in Appendix) of breakthrough infections reported in 3 studies (gray/black dots). Data from 2 mRNA vaccine studies of mRNA-1273 (A) and BNT162b2 (B), and the adenoviral vector vaccine ChAdOx1 nCoV19 (C, D) are shown. Lower opacity dots indicate fewer persons with neutralization titers in that range. Also shown in each panel are modelled protection curves showing the relationship between individual neutralizing antibodies and protection estimated in each breakthrough-infection study. Note: Breakthrough-infection data of BNT162b2 vaccinees were generously supplied by the authors of reference (*5*). The data were unavailable for the other 2 studies and were extracted from the original manuscripts; extraction of data from Gilbert et al. (*4*) was conducted manually and may be less reliable than that of the other studies (Appendix). ID_50_, 50% infectious dose; ID_80_, 80% infectious dose.

The analysis above does not allow direct visualization or comparison of the fit of the data from breakthrough infection to the data from the vaccine-comparison study. We developed a method for estimating unadjusted protection at different neutralization levels from the breakthrough-infection data (Appendix), which also enables inclusion of data from a third breakthrough-infection study of BNT162b2 (Pfizer-BioNTech, https://www.pfizer.com) vaccinees (*5*). We show data from the 3 breakthrough-infection studies compared with the vaccine-comparison approach (normalized for the mean vaccinee titer in each study) (Figure 3). Data from the breakthrough-infection studies show remarkable agreement with the vaccine-comparison model (within the neutralization ranges for which sufficient data were available for each breakthrough-infection study), despite the fundamentally different data, assays, and approaches used to estimate protection curves in each study. Furthermore, after alignment to the GMT of each vaccine group, we can use the underlying distribution in neutralizing antibody titers along with the protection curves from each of these studies to predict the overall vaccine efficacy for existing vaccines (as has been done for the Khoury et al. model [*2*)]) (Appendix Methods). That approach reveals good agreement between all models and the observed data, at least in the ranges where data were available to parameterize the models (Appendix Figure 1). This approach provides cross-validation of the protection curves but also provides a lesson that all models should be used cautiously outside the ranges of the data over which they were developed.

## Using the Protection Curve

### Immunobridging to Predict Vaccine Efficacy

For vaccine development, an immune correlate to predict the efficacy of a novel vaccine without the need for large and expensive phase 3 efficacy trials would greatly accelerate the approval of novel vaccines (*13*). Similarly, for incorporating novel SARS-CoV-2 variant immunogens, being able to use surrogate measures to predict vaccine efficacy would be helpful. On a public health level, information about neutralization of new variants as they arise and predicting likely population immunity to them would help with predicting future infection risk. In addition, predicting changes in vaccine efficacy with immunity waning and in cohorts with lower neutralization titers after vaccination (e.g., in elderly or immunocompromised persons) could provide information about the need for boosting and other immune protective strategies (*12*).

If a standardized neutralization assay were widely used, it would, in principle, be possible to offer a globally applicable GMT neutralization titer (threshold) associated with a given level of protection, which regulators and vaccine developers could use as a target when assessing and approving vaccines (e.g., as the hemagglutination inhibition titer provides for influenza infection). However, the lack of assay standardization means that no such threshold in international units can be determined that is broadly applicable across different neutralizing antibody assays. Alternatively, regulators have signaled that immunobridging studies, which compare the immunogenicity of new vaccines with that of existing vaccines (for which efficacy has previously been determined) should be conducted (*14*,*15*). That is, vaccine developers need to identify a suitable existing vaccine for comparison and determine the noninferiority or superiority margins relative to these vaccines in a randomized controlled trial (i.e., how much higher neutralization titers are required to be or how much lower titers are permitted to be compared with existing vaccines). The protection curves reported so far (*2*–*4*) can be used to define the parameters of these noninferiority or superiority trials. For example, using the vaccine-comparison model derived by Khoury et al. (*2*) (Figure 1, panel C), we can estimate the noninferiority or superiority margins to existing vaccines that would provide >80% efficacy against ancestral virus (Appendix Table 2, Figure 2). If mRNA-1273 or BNT162b2 are used as comparator vaccines, finding a noninferiority margin of 0.44-fold of the GMT observed in mRNA-1273 vaccinees or 0.54-fold of the GMT observed in BNT162b2 vaccinees would provide high confidence that the candidate vaccine has >80% efficacy (against ancestral virus). Using ChAdOx1 (with 4-week spacing of doses) as a comparator, we found that a superiority margin of 2.6-fold of GMT compared with ChAdOx1 vaccinees would provide similarly high confidence of >80% vaccine efficacy. Of note, those margins are in strong agreement with the lower 95% CIs predicted in the breakthrough-infection studies (Figure 2), which would predict that a candidate vaccine that induced 0.44-fold of the GMT for mRNA-1273 vaccinees would be expected to have an efficacy of >85% (either of the 2 neutralization assays reported in that study, on the basis of the reported lower 95% CI) and that a margin of 2.6-fold of the mean ChAdOx1 titer would predict an efficacy of >76% (the lower 95% CI of Feng et al. models do not reach 80% in all cases (Figure 2; Appendix) (*3*,*4*). The consensus of these 3 studies provides strong support for using noninferiority or superiority margins in future immunobridging studies.

### Identifying Protective Thresholds for Individual Persons

A second goal for the study of protective thresholds is to identify a protective titer for clinical use, that is, a simple blood test for clinically relevant antibody level to indicate if a person is likely to have good protection from COVID-19. The studies that have defined the relationship between neutralization titer and vaccine efficacy have not been designed for, and are not primarily concerned with, defining such a threshold because they deal only with estimates of vaccine efficacy at a population level. Furthermore, individual predictions from population statistics can be fraught with difficulty. Unfortunately, the term “threshold” gives the impression that there might be an antibody level above which one is fully protected (and below which one is susceptible). However, the shapes of the protection curves (Figure 2) make it clear that there is a gradient of risk at different neutralization titers. Moreover, the between-run variability of assays is typically large enough that the uncertainty in the neutralization titer estimated for an individual serum sample is sufficient to lead to wide confidence intervals for the predicted protection for that person (Appendix). For example, when typical duplicate-well and 2-fold serum dilution neutralizing assay designs are used (*16*,*17*), a person with a neutralizing antibody titer at exactly the level associated with 50% protection would have 95% CIs on the estimated protection, ranging from 15% to 85% protection (Appendix), although that range will depend on the precision of a particular assay. It is worth noting that these are estimates of protection from symptomatic SARS-CoV-2 infection (the primary outcome of the studies analyzed), and protection against severe outcomes is achieved at lower neutralization titers (*2*). Together, the wide CIs when estimating individual neutralization titers and the standardization between different serologic assays are major limitations for ability to accurately assess individual neutralizing antibody titers and predict individual protection.

## Discussion

Predicting vaccine efficacy or a clinically useful threshold of protection against COVID-19 would be a major advance. The in vitro neutralization titer has been demonstrated by multiple studies to be well correlated with vaccine efficacy and with a person’s protection from symptomatic SARS-CoV-2 infection (*2*–*6*,*12*; D.H. Khoury et al., unpub. data). The 4 studies that found significant relationships between neutralization titers and vaccine efficacy used different methods (*2**–**5*), and data from different clinical trials with neutralization data assessed across a range of neutralization assays. Those factors may all contribute to apparent discrepancies between the relationships reported in each study. However, we show that after centering the data from each study on the GMT of the vaccine used in each study, the 4 studies converge on a common prediction of the relationship between neutralization and protection against infection (within the bounds of data available within each study). The agreement of these studies strongly supports the use of neutralizing antibody titers to predict the efficacy of new vaccines or vaccine efficacy against new variants (assuming the fold drop in neutralization titer for the variant can be estimated). Although neutralizing antibody levels are a clear correlate of protection, identifying a protective threshold applicable to a serologic test is more challenging, in part because no such threshold exists, but instead, there is a gradient of vaccine efficacy that increases with neutralization. Furthermore, significant challenges to defining a particular threshold at which a person’s neutralization titer might be deemed to provide high protection from COVID-19 include the diversity of assays used to measure neutralization, the difficulty in translating neutralization levels between assays, the constant emergence of new and more escaped variants, and the uncertainties of estimating individual neutralization titers. 

An additional major challenge is adapting assays (and protection curves) to deal with neutralization of current and future SARS-CoV-2 variants. The studies discussed in this analysis primarily deal with neutralization of and protection from the ancestral SARS-CoV-2 strain because the breakthrough-infection data and vaccine efficacy data in most studies was from phase 3 clinical trials (*2*–*4*), which studied infection within the first few months after vaccination and which were mainly conducted before variants of concern had a major foothold, except for the Bergwerk et al. study (*5*), which was conducted during in the Alpha-dominant period. It would be ideal to be able to adapt each model of immune correlates to test its ability to predict protection against variants of concern. However, until recently, only the vaccine-comparison model has been extended to analyze protection against SARS-CoV-2 variants (*12*,*18*,*19*; D. Cromer et al., unpub. data, https://www.medrxiv.org/content/10.1101/2022.06.09.22275942v1), although a recent study has begun to explore this question by using a breakthrough-infection approach (*20*). The work on the vaccine-comparison model approach has so far shown that this model, which was originally calibrated on data for ancestral SARS-CoV-2 infections, can also be used to predict vaccine effectiveness against SARS-CoV-2 variants and after boosting, as long as one adjusts for the drop in neutralization titers to the variants and rise in neutralization after boosting (*12*,*18*,*19*; D. Cromer et al., unpub. data). However, the need to standardize neutralization assays for SARS-CoV-2 variants presents an ongoing challenge.

In vitro neutralizing antibody titers against SARS-CoV-2 present a clear correlate of protection from symptomatic SARS-CoV-2 infection. Studies of passive administration of neutralizing monoclonal antibodies in animals and humans support that neutralizing antibody titers are a mechanistic correlate of protection (*21*–*23*). Indeed, a recent study comparing protective titers in prophylactic and therapeutic studies suggests that the protective titers may be very similar (E. Stadler et al., unpub. data, https://www.medrxiv.org/content/10.1101/2022.03.21.22272672v2). Neutralizing antibody levels are also correlated with protection from severe SARS-CoV-2 infection (*2*). 

In conclusion, our findings show that the different COVID-19 correlate of protection studies, which seemingly report different thresholds of protection, have strong agreement. However, other immune responses may also play a substantial role in protection against progression from symptomatic to severe SARS-CoV-2 infection. The agreement across multiple studies of the relationship between neutralizing antibodies and efficacy against COVID-19 can be useful for planning future vaccine use, determining population immunity, and reducing the global effects of the COVID-19 pandemic.

Appendix Supplementary methods and analyses for study of correlates of protection for SARS-CoV-2 infection**.**
